# A Novel Bacitracin-like Peptide from Mangrove-Isolated *Bacillus paralicheniformis* NNS4-3 against MRSA and Its Genomic Insights

**DOI:** 10.3390/antibiotics13080716

**Published:** 2024-07-30

**Authors:** Namfa Sermkaew, Apichart Atipairin, Thamonwan Wanganuttara, Sucheewin Krobthong, Chanat Aonbangkhen, Yodying Yingchutrakul, Jumpei Uchiyama, Nuttapon Songnaka

**Affiliations:** 1 School of Pharmacy, Walailak University, Thasala, Nakhon Si Thammarat 80160, Thailand; namfa.se@wu.ac.th (N.S.); apichart.at@wu.ac.th (A.A.); thamonwan.wa@wu.ac.th (T.W.); 2 Drug and Cosmetics Excellence Center, Walailak University, Thasala, Nakhon Si Thammarat 80160, Thailand; 3 Center of Excellence in Natural Products Chemistry (CENP), Department of Chemistry, Faculty of Science, Chulalongkorn University, Bangkok 10330, Thailand; sucheewin.k@chula.ac.th (S.K.); chanat.a@chula.ac.th (C.A.); 4 Center of Excellence on Petrochemical and Materials Technology, Chulalongkorn University, Pathumwan, Bangkok 10330, Thailand; 5 National Center for Genetic Engineering and Biotechnology, National Science and Technology Development Agency, Pathum Thani 12120, Thailand; yodying.yin@biotec.or.th; 6 Department of Bacteriology, Graduate School of Medicine, Dentistry and Pharmaceutical Sciences, Okayama University, Okayama 700-8558, Japan; uchiyama@okayama-u.ac.jp

**Keywords:** antimicrobial peptide, antimicrobial resistance, *Bacillus paralicheniformis*, bacterial genome, biosynthetic gene cluster, mangrove, marine bacteria, mass spectrometry, MRSA, NNS4-3

## Abstract

The global rise of antimicrobial resistance (AMR) presents a critical challenge necessitating the discovery of novel antimicrobial agents. Mangrove microbes are valuable sources of new antimicrobial compounds. This study reports the discovery of a potent antimicrobial peptide (AMP) from *Bacillus paralicheniformis* NNS4-3, isolated from mangrove sediment, exhibiting significant activity against methicillin-resistant *Staphylococcus aureus* (MRSA). The AMP demonstrated a minimum inhibitory concentration ranging from 1 to 16 µg/mL in the tested bacteria and exhibited bactericidal effects at higher concentrations. Structural analysis revealed a bacitracin-like configuration and the peptide acted by disrupting bacterial membranes in a time- and concentration-dependent manner. The AMP maintained stability under heat, proteolytic enzymes, surfactants, and varying pH treatments. The ten biosynthetic gene clusters (BGCs) of secondary metabolites were found in the genome. Detailed sequence comparison of the predicted bacitracin BGC indicated distinct DNA sequences compared to previously reported strains. Although the antibiotic resistance genes were found, this strain was susceptible to antibiotics. Our findings demonstrated the potential of *Bacillus paralicheniformis* NNS4-3 and its AMP as a promising agent in combating AMR. The genetic information could be pivotal for future applications in the healthcare industry, emphasizing the need for continued exploration of marine microbial diversity in drug discovery.

## 1. Introduction

Antimicrobial resistance (AMR) is a current significant issue in global public health. Its severity has heightened due to the excessive and inappropriate use of antimicrobial agents in humans, animals, and plants and insufficient infection prevention measures. This contributes to the emergence of new and resistant microorganisms in the environmental habitat. In 2019, there were 1.27 million deaths attributable to bacterial antimicrobial resistance. Unfortunately, methicillin-resistant *Staphylococcus aureus* (MRSA) was among the top pathogens responsible for mortality associated with antimicrobial resistance [1]. The prediction indicates a potential increase to 10 million annual deaths by 2050 with a projected economic impact of USD 100 trillion [2]. Due to the natural evolutionary process, organisms develop genetic mutations to withstand lethal selection pressures. AMR is inevitable and bacteria will inherently adapt and exhibit defensive mechanisms as long as antibacterial agents are utilized against them.

*Staphylococcus aureus* (*S. aureus*) exhibits the capacity to cause significant infections and resistance to various antibacterial medications. *S. aureus* has the ability to adapt to both its human host and the environment, becoming resistant to antibiotics. It is a prominent causative microorganism of endocarditis, bacteremia, osteomyelitis, and skin and soft tissue infections. Each of these conditions has the potential to result in sepsis eventually. After the introduction of the first semi-synthetic anti-staphylococcal penicillins around 1960, the emergence of MRSA was observed within 1 year of their clinical application [3]. Resistance to methicillin in *S. aureus* is due to the existence of the *mec*A (or *mec*C) gene, which is responsible for encoding the penicillin-binding protein PBP2a. This protein exhibits reduced affinity for semi-synthetic penicillins [4]. The World Health Organization (WHO) has categorized MRSA as a high-priority concern, highlighting the urgent need to develop antimicrobial agents for clinical situations. The golden era of antibiotics is fading due to the lack of discovery of new classes of antibiotics leading to the transition into a “post-antibiotics” era. This transition means a lack of effective drugs for antimicrobial-resistance treatment. Therefore, the discovery of new antimicrobial agents for treating infectious diseases caused by MRSA is very urgent.

Antimicrobial peptides (AMPs) are present in every kingdom of life and they are an essential element of host defense mechanisms. AMPs represent a category of short-chain peptides, typically comprising amino acids ranging from 12 to 50 residues. Discovered in 1939 from *Bacillus brevis*, gramicidin was the first AMP exhibiting antibacterial effectiveness against various Gram-positive bacteria in clinical uses [5,6]. AMPs are commonly amphiphilic and possess a high cationic nature. The physicochemical properties of AMPs play a crucial role in their antimicrobial activity. Certain AMPs show the ability to kill bacteria without affecting membrane integrity. These AMPs directly enter bacterial cells, disrupting crucial cellular functions, such as DNA replication, transcription, translation, protein folding, and the process of cell division [7]. The drug resistance to AMPs resulting from genetic mutations has a low probability due to the difficulty of cell membrane alteration and the versatile mechanisms of AMPs. Consequently, the development of drug resistance by bacteria is more difficult for AMPs compared to antibiotics [8]. Numerous peptides sourced from the marine environment have been isolated and proven to be promising compounds for drug candidates due to their unique molecular mechanisms of action [9]. Interestingly, the search for potential bacteria capable of producing antibiotics has transitioned from land-based strains to marine microorganisms. The high-stress and complicated marine environments, such as salt concentrations, pressure, temperature, oxygen concentrations, and radiation exposure, stimulate bacteria to produce compounds distinct from those found in terrestrial counterparts [10].

This research aimed to derive potential AMP-producing bacteria from mangrove sediment, along with their genomic information. The obtained AMP was purified and characterized before determining its antibacterial activity and chemical structure.

## 2. Results

### 2.1. Exploration of Antimicrobial-Producing Bacterial Isolates from Mangrove Sediments

Sediment samples were collected from five different locations in the mangrove area in the Pak Banang district, Nakhon Si Thammarat, Thailand. The pH values of the sediments ranged from 6.26 to 8.00 and the salinity of the sediments ranged from 9.00 to 10.00 ppt. All colonies obtained from Mueller Hinton (MH) agar, Zobell Marine (ZM) agar, and Starch Casein (SC) agar were screened for anti-MRSA activity against MRSA strain 2468 using the soft agar overlay method. The isolates active against MRSA were verified by subculturing them in a liquid medium before collecting the cell-free supernatant (CFS). The spectrum of antibacterial activity was evaluated by agar well diffusion assay against various pathogenic bacteria, such as *S. aureus* TISTR 517 and MRSA strains 142, 1096, and 2468. Only four isolates from ZM agar exhibited antibacterial activity against different pathogens. It was observed that NNS4-5-2 isolate demonstrated antibacterial activity against MRSA but not against *S. aureus*. Nevertheless, three isolates (NNS2-1, NNS4-2, and NNS4-3) exhibited inhibitory effects against *S. aureus* TISTR 517, as well as MRSA strains 142, 1096, and 2468. Therefore, three isolates demonstrated antibacterial activity against both antibiotic-susceptible and antibiotic-resistant strains of *S. aureus*. Notably, the NNS4-3 isolate exhibited the greatest diameter of inhibition zone against MRSA strains compared to the other isolates. Its antibacterial activity against three strains of MRSA was superior to cefoxitin and comparable to vancomycin (Table 1). Consequently, this investigation focused on the antibacterial components produced by the NNS4-3 isolate to explore the significant findings of its genome and the antimicrobial active compound.

### 2.2. Kinetics of Antibacterial Component Production of NNS4-3

**Figure 1:**
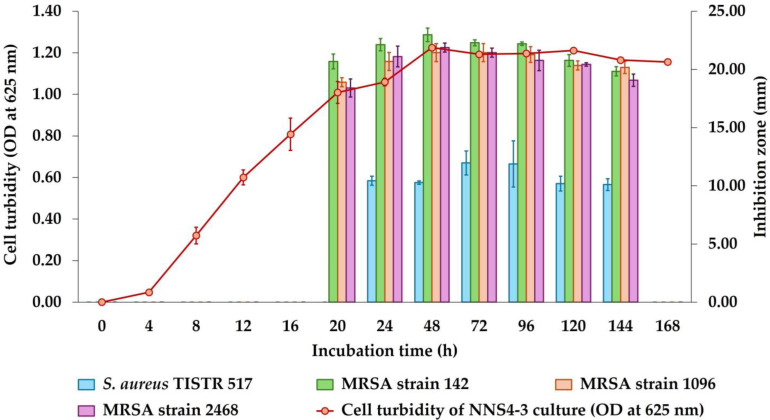
Growth curve and production kinetics of antibacterial components of NNS4-3.

The incubation of NNS4-3 was performed from 1 to 7 days and the growth curve was plotted by measuring the OD at 625 nm while the antibacterial activity was determined after collecting CFSs at each time interval. The maximum activity of CFSs derived from NNS4-3 was observed at 48 h with inhibition zones of 22.97 ± 0.58, 21.45 ± 0.78, and 21.87 ± 0.38 mm against MRSA strains 142, 1096, and 2468, respectively. The inhibitory effect against *S. aureus* TISTR 517 was at its maximum at 72 h with an inhibition zone of 11.97 ± 1.03 mm. However, a decrease in antibacterial activity against four strains of *S. aureus* was observed from 48 h to 144 h of cultivation. The antibacterial activity disappeared when the incubation reached 168 h. The antibacterial components produced by NNS4-3 exhibited no significant difference in antibacterial activity against all MRSA strains at all time intervals (Figure 1). However, the antibacterial activity against antibiotic-susceptible *S. aureus* was significantly lower than that against MRSA strains across all time intervals.

### 2.3. Purification of the Active Antibacterial Components

**Figure 2:**
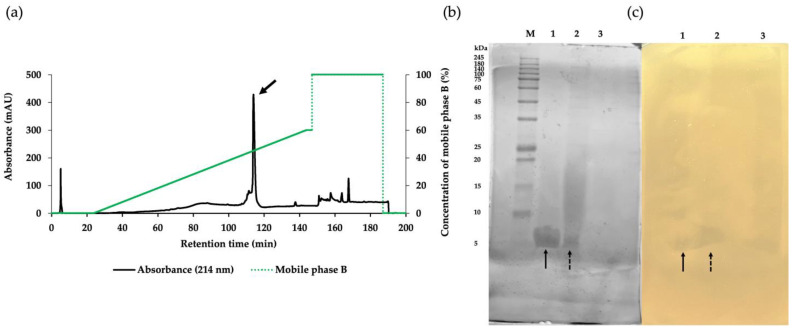
The purification of the AMP derived from NNS4-3 separated by RPC with gradient elution of mobile phase B (green solid line) showed that the major peak (arrow) was the active fraction (**a**). SDS-PAGE with 15% gel was performed for the characterization of protein bands stained with coomassie brilliant blue (**b**). The active fraction from each purification step, as followed by RPC (Lane 1), protein precipitation (Lane 2), and CFS collected from NNS4-3 culture (Lane 3), was electrophoresed and compared with the protein marker (Lane M). The protein bands exhibiting antibacterial activity were observed as an inhibition zone when overlaid with soft agar containing MRSA strain 2468 (**c**). The correlation between protein bands in the stained gel and the inhibition zone in the soft-agar overlaid gel in Lane 1 (solid arrow) and Lane 2 (dashed arrow) was found at a similar position.

The crude product of antimicrobial components in the CFS of NNS4-3 was purified using ammonium sulfate precipitation and reversed-phase chromatography (RPC). Each fraction obtained from the purification steps was tested for antibacterial activity against MRSA strain 2468 using the agar well diffusion method. The CFS exhibited antibacterial activity against the MRSA strain 2468. The active precipitate was obtained at 75% saturation with ammonium sulfate. The collected precipitate was dissolved and desalted by dialysis in 0.1% trifluoroacetic acid (TFA). The dialysate was further purified by RPC to confirm its antibacterial activity. The active RPC fraction eluted with 50% buffer B exhibited intense absorbance at 214 nm, indicating the presence of peptides (Figure 2a). The purification efficacy and molecular weight of the active fractions were estimated by sodium dodecyl sulfate-polyacrylamide gel electrophoresis (SDS-PAGE). A single active band in Lane 1 indicated the effective purification process while Lanes 2 and 3 from ammonium sulfate precipitation and CFS, respectively, showed partial purification (Figure 2b). The electrophoresed gel showed an intense coomassie-blue-stained band between 5 and 10 kDa when compared to the protein marker in Lane M. To locate the active protein band, the other half of the gel was overlaid with MRSA strain 2468 soft agar and the clear zones revealed a molecular weight between 5 and 10 kDa (Figure 2c). The characterization of the protein band demonstrated that the purification procedure effectively concentrated the target protein and excluded other proteins, as indicated by the darkening of the active band and fading of others. The purification procedure was evaluated using a purification data sheet and the results showed a 22.65-fold increase in purification power compared to the initial crude extract with a 9.76% yield of the active peptide (Table 2).

### 2.4. De Novo Amino Acid Sequence of the Purified AMP and Secondary Structure Determination

**Figure 3:**
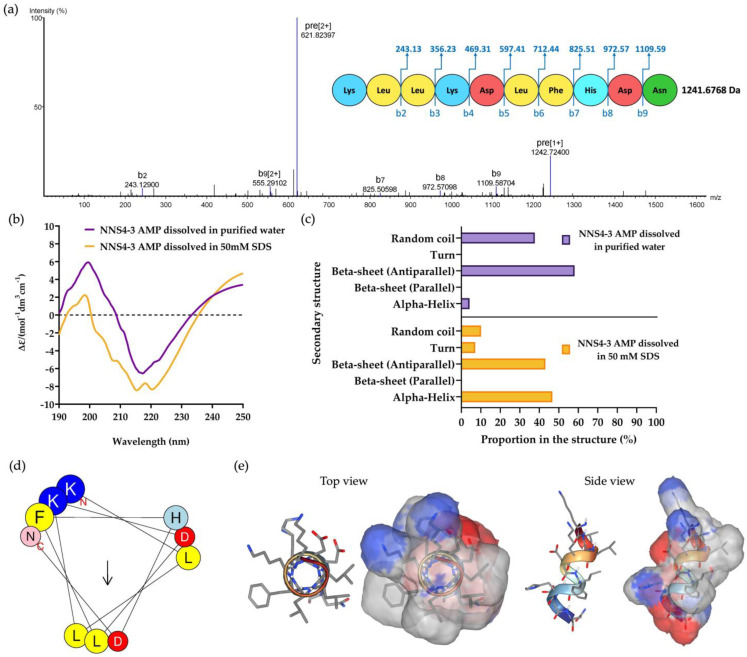
The mass spectrum of NNS4-3 AMP showed amino acid sequence by de novo sequencing with b-ion and mass detection with positive mode (**a**). The secondary structure of the determined amino acid sequence was analyzed by CD spectroscopy. The CD spectrum from 190 to 250 nm was observed when NNS4-3 AMP was dissolved in purified water or 50 mM SDS solution (**b**). The determination of AMP secondary structure components using CD spectra was analyzed by the BeStSel method via a web-based service (**c**). The amino acid arrangement of an alpha-helix structure of the peptide was predicted by HELIQUEST (the arrow indicates the hydrophobic face of the peptide) (**d**). The 3D model with the molecular surface was predicted using the web-based structure prediction from PEP-FOLD4. The top view and side view display the molecular surface area with the positive electrostatic potential area (indicated in blue), negative electrostatic potential area (indicated in red), and hydrophobic area (indicated in grey) (**e**).

The purified NNS4-3 AMP from RPC was collected and subsequently subjected to amino acid sequencing by tandem mass spectrometry. The de novo algorithm was used to predict the amino acid sequence from b-ion fragmentation. The molecular weight of the sequenced peptide was 1241.6768 Da (single protonated molecule in mass spectrogram reported 1242.72400 Da). The AMP was composed of 10 amino acid residues and sequenced as Lys-Leu-Leu-Lys-Asp-Leu-Phe-His-Asp-Asn with an average local confidence (ALC) score of 76% (Figure 3a). The prediction of the physicochemical properties of the peptide was performed by ProtParam on the Expasy server. The results revealed that the peptide had a theoretical pI of 6.75. The peptide contained positively charged amino acids due to two lysines and negatively charged amino acids due to two aspartic acids. The total net charge was +0.25 at pH 7.4. The grand average of hydropathy (GRAVY) value of the peptide was −0.730, predicted by hydrophobic amino acids, which were leucine and phenylalanine, and hydrophilic amino acids, which were lysine, aspartic acid, histidine, and asparagine.

Circular dichroism (CD) spectroscopy was used to determine the secondary structure of the NNS4-3 AMP. The secondary structure of the AMP was studied when the AMP was dissolved in different solvents (purified water and 50 mM sodium dodecyl sulfate; SDS). Purified water was used as a solvent for studying the AMP secondary structure in its native conformation, whereas 50 mM SDS was used to form micelles for studying the interaction of the AMP, which simulates the negatively charged bacterial cell membrane environment [11]. The CD results showed a high positive band at 190–230 nm with a magnitude of 200 nm and a negative band at 205–240 nm with a magnitude of 220 nm in both solvent systems. The results indicated that alpha-helix and beta-sheet conformations were found in the structure of the NNS4-3 AMP. When comparing the use of purified water and 50 mM SDS as solvents, the results showed that the positive band at 190–230 nm decreased in magnitude, whereas the negative band at 205–240 nm increased in magnitude when the AMP was dissolved in SDS. The change in magnitude indicated a transformation in the AMP structure to an alpha-helix conformation when dissolved in the SDS solution (Figure 3b). The components of the AMP secondary structure were calculated using CD spectra by the BeStSel web-based service. Different components of structural conformation were found when using purified water and 50 mM SDS solution as solvents. The AMP dissolved in purified water revealed that the major component, the beta-sheet conformation with an antiparallel direction, accounted for 58.1% while the alpha-helix conformation was 4.2% and the random coil conformation was 37.7%. In contrast, when the AMP was dissolved in 50 mM SDS, the major component of structural conformation changed to 46.7% of the alpha-helix conformation, along with 10.1% of random coil conformation, while the beta-sheet with the antiparallel direction was reduced to 43.1% (Figure 3c). The component changes of AMP secondary structure when dissolved in 50 mM SDS suggested that the random coil conformation of the NNS4-3 AMP structure was converted to an alpha-helix conformation by the interaction of SDS micelles through electrostatic and hydrophobic interactions between the AMP and the surface of SDS micelles. The amino acid arrangement of NNS4-3 AMP in an alpha-helix conformation was predicted by a helical wheel from the HELIQUEST web-based service. The predicted helical wheel showed three groups of amino acid arrangement, namely, positively charged, negatively charged, and hydrophobic areas (Figure 3d). For more visualization of the 3D molecular surface area of NNS4-3 AMP, the secondary structure was predicted by PEP-FOLD4. The predicted molecular model of NNS4-3 AMP showed a polar region localized around the N-terminus and C-terminus, indicated by blue (positive electrostatic potential) and red (negative electrostatic potential) colors, while the hydrophobic region indicated by grey color was in the middle of the structure (Figure 3e). The predicted 3D structure of NNS4-3 AMP was consistent with the helical wheel prediction.

### 2.5. Determination of the Antimicrobial Activity of NNS4-3 AMP by the Microdilution Method

The microdilution method was performed to determine the antibacterial activity of the purified AMP of NNS4-3 compared to the standard antibiotics. Minimum inhibitory concentrations (MICs) were different between antibiotic-susceptible strains and resistant strains. *S. aureus* TISTR 517 was inhibited by 16 µg/mL. This concentration was 16-fold higher than the MIC value for three resistant strains (MRSA strains 142, 1096, and 2468) at 1 µg/mL. NNS4-3 AMP required 4-fold the amount of the MIC to exhibit the bactericidal effect against all tested strains. Vancomycin exhibited an inhibitory effect with a killing action because the MIC and MBC were equal. In contrast, cefoxitin showed equal MIC against *S. aureus* TISTR 517 but it was ineffective against all resistant strains (Table 3).

### 2.6. Scanning Electron Microscopic Studies

**Figure 4:**
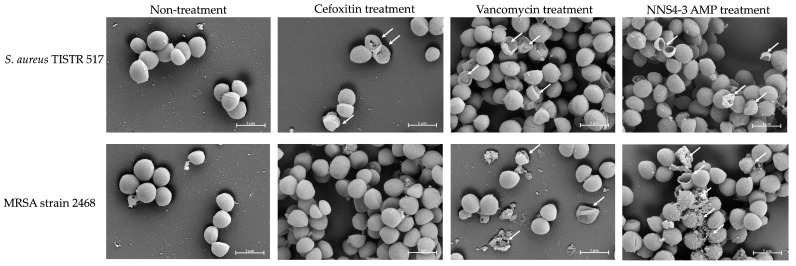
Photos captured using SEM at 20,000× magnification showed the morphological changes of *S. aureus* TISTR 517 and MRSA strain 2468 under antibiotics (vancomycin and cefoxitin) and 1× MIC of NNS4-3 AMP treatment. The non-treatment condition was used as a negative control. The arrow indicates the membrane disruption.

Scanning electron microscopy (SEM) was employed to investigate the morphological alterations and membrane damage in *S. aureus* and MRSA caused by AMPs and standard antibiotics. After exposure to the NNS4-3 AMP, cell walls and cell membranes of pathogenic bacterial cells appeared to rupture and pore formation was observed (Figure 4, indicated by an arrow). The results indicated that the pathogens were sensitive to AMPs at 1× MIC at 18 h of incubation. *S. aureus* TISTR 517 showed cellular lysis when treated with cefoxitin, vancomycin, and NNS4-3 AMP. In the non-treatment condition, the cell membranes were intact with smooth surfaces and regular shapes. In contrast, MRSA strain 2468 showed good tolerance to cefoxitin treatment due to its resistant nature and that was similar to intact cell characteristics under non-treatment conditions. Vancomycin exhibited the potential to kill MRSA cells, as indicated by the irregular shape observed. The rupture of cell membranes indicated that the treated cells could not maintain their regular shape due to the interference with the D-alanyl-D-alanine moiety in cell wall synthesis by vancomycin. Interestingly, the antibacterial activity of NNS4-3 AMP resulted in the membrane disruption and pore formation that was indicated by the extrusion of the cytoplasm. The irregular shape of the treated cells and membrane damage, revealed by the rough surface of the cellular membrane, indicated the destabilization of cell membranes caused by AMPs.

### 2.7. Killing Kinetics of the AMP Isolated from NNS4-3

**Figure 5:**
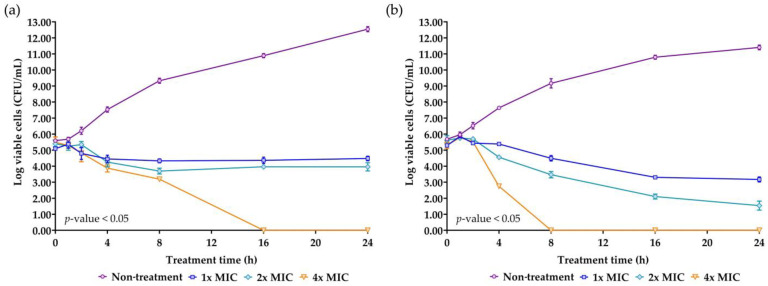
Killing kinetics of NNS4-3 AMP. *S. aureus* TISTR 517 (**a**) and MRSA strain 2468 (**b**) were incubated with 1× (☐), 2× (◇), and 4× MIC (▽) of NNS4-3 AMP compared to the non-treatment group (◯). Significant differences in viable cell reduction at each time point (*p*-value < 0.05) were analyzed by two-way ANOVA.

Time-kill kinetics is a crucial parameter for evaluating the efficacy and the mode of action of the AMP. The concentrations of NNS4-3 AMP at 1× MIC, 2× MIC, and 4× MIC were used to treat *S. aureus* TISTR 517 and MRSA strain 2468. A significant reduction in survival cells was observed at 4 h of treatment compared to the non-treatment condition in both strains. The antimicrobial effect at the concentration of 1× MIC and 2× MIC against *S. aureus* TISTR 517 caused the survival cells to initially decline at 4 h and survival cells continued to decrease until 8 h of treatment. After that, the survival cell numbers slightly increased but the number of survival cells remained lower than the initial count until 24 h. The findings indicated that even after 24 h, NNS4-3 AMP at 1× MIC and 2× MIC did not completely kill *S. aureus* TISTR 517. The time-kill curve showed a log reduction rate of the survival cells at 1× MIC and 2× MIC of 0.028 and 0.061 logCFU/h, respectively. However, at a concentration of 4× MIC, the AMP continuously killed *S. aureus* TISTR 517 with the highest log reduction rate of 0.339 logCFU/h. In addition, the concentration at 4× MIC completely eradicated *S. aureus* TISTR 517 at 16 h of treatment. NNS4-3 AMP showed stronger inhibition to MRSA strain 2468 compared to *S. aureus* TISTR 517. The log reduction rates of MRSA strain 2468 treated with the AMP at the concentrations of 1× MIC, 2× MIC, and 4× MIC were 0.115, 0.190, and 0.757 logCFU/h, respectively. Furthermore, the viable cells were completely eliminated within 8 h after treatment with 4× MIC of AMP (Figure 5). The different concentrations of NNS4-3 AMP between 1× MIC, 2× MIC, and 4× MIC revealed different reduction trends when the treatment time reached 16 h and 4 h against *S. aureus* TISTR 517 and MRSA strain 2468, respectively. The inhibitory concentrations were consistent with MIC and MBC determinations. The mode of action of AMP could be proposed in a concentration- and time-dependent manner against both pathogens. However, the time-killing curve provided information that wild-type and antibiotic-resistant strains were differently sensitive to the NNS4-3 AMP.

### 2.8. Stability Determination of NNS4-3 AMP under Various Conditions: Temperature, Proteolytic Enzymes, Surfactants, and Acid-Base Treatment

The stability of the NNS4-3 AMP under various conditions is shown in Table 4. Thermal stability studies revealed that the antibacterial activity of the AMP against MRSA strain 2468 was maintained at 37 °C for up to 12 h. The AMP retained its antibacterial activity at 60 °C for up to 6 h of incubation, whereas a significant reduction was observed at 12 h. The activities decreased to half potency when the temperature was above 80 °C after 6 h and 12 h of incubation. The antibacterial activity of the AMP decreased to approximately 73% when treated at 100 °C for 1 h and the activity completely diminished to 0% over subsequent incubation periods. For more thermal-stressed conditions, autoclaving was performed. AMP activity was reduced to 61% after autoclaving for 15 min. The activity of the AMP completely disappeared after autoclaving for 30 min. The tolerance of the AMP to proteolytic enzymes showed that NNS4-3 AMP resisted three different proteolytic enzymes (proteinase K, trypsin, and α-chymotrypsin) for up to 12 h of incubation. The results showed that the overall activity of the AMP was more than 90%. Surfactants above the critical micelle concentration were used in the experiment as the interferences of AMP activity. SDS and Triton X-100 affected the AMP activity differently. SDS, the ionic surfactant, reduced the antibacterial activity of the AMP; however, the non-ionic surfactant Triton X-100 did not affect the activity of the AMP. A decrease in AMP activity after treatment with surfactants did not depend on the incubation time. There was no significant difference in the activity of the AMP incubated at different physiological pHs, including pHs 1.2, 4.5, 6.8, and 7.4, when compared to the non-treated sample, which was the AMP dissolved in purified water. However, the activity of the AMP decreased with increasing incubation time. The stability studies of the AMP revealed that it was tolerant of different environmental factors, maintaining more than 90% activity. However, temperatures exceeding 80 °C or exposure to an ionic surfactant should be considered for assessing the robustness of its activity.

### 2.9. Phenotypic Characterization of NNS4-3

**Figure 6:**
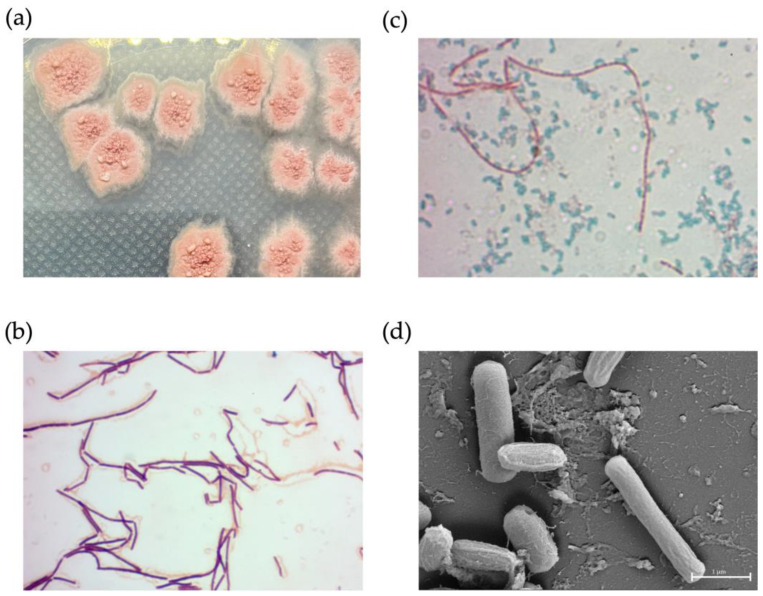
The physical characteristics of NNS4-3. A single colony morphology displayed on ZM agar (**a**), Gram-stained vegetative cells (**b**), and malachite-green-stained endospores (**c**) were visualized under a light microscope at 1000× magnification. High-resolution scanning electron micrography revealed vegetative and endospore morphology at 20,000× magnification (**d**).

NNS4-3 displayed a single colony with a rough surface and filamentous border. After 2 days of culture, the colony began to produce red pigment (Figure 6a). Light microscope observations showed Gram-positive streptobacilli (Figure 6b) and the endospores were stained with malachite green after 3 days of culture (Figure 6c). The dimensions of a single cell were 0.33–0.56 µm in width and 2.44–2.57 µm in length. When cells were forming endospores, cellular dimensions reduced in length to 2.04–2.16 µm and the width increased to 0.63–0.78 µm. The spores exhibited a wrinkled morphology due to cell membrane shrinkage; therefore, the cell dimensions were reduced to be compact with a width of 0.48–0.58 µm and a length of 1.03–1.12 µm (Figure 6d).

### 2.10. Genome Insights for Coding Sequence Annotation and Genome-Based Phylogenetic Tree Construction

**Figure 7:**
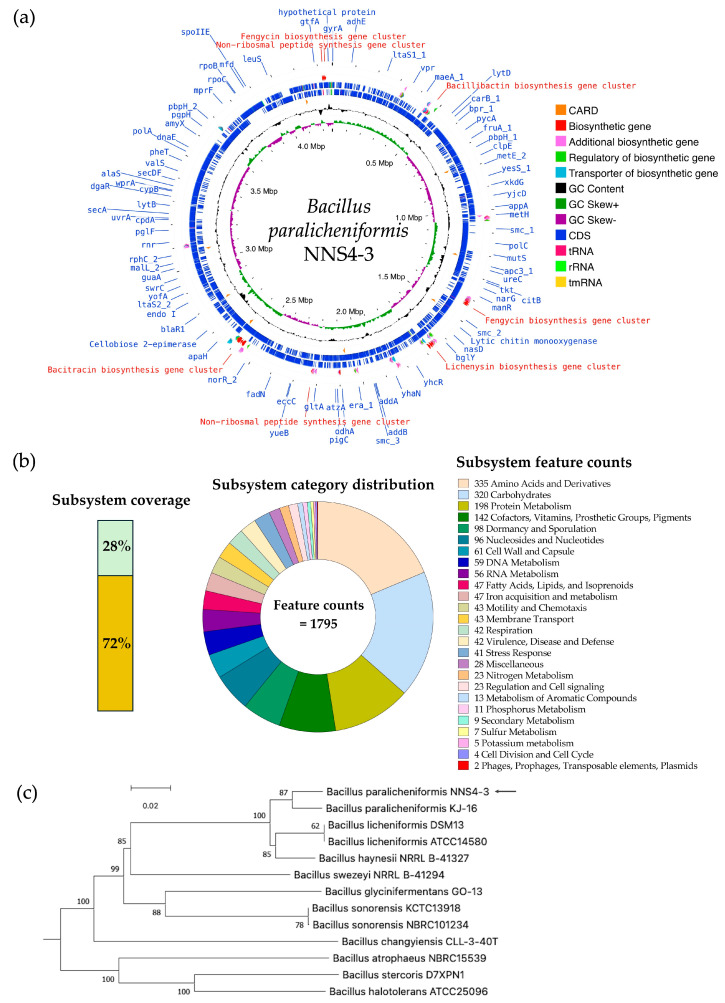
The genome insight of *Bacillus paralicheniformis* NNS4-3 is displayed by a circular genome map visualized by Proksee (**a**). RAST provided the information on the cellular machinery that was predicted by the coding sequences used subsystem technology for functionalization and defined metabolic pathways of organisms (**b**). The GBDP method was performed for phylogenies determination and a query genome (arrow) was predicted against microorganism genomes in the TYGS database for the closest phylogenetic relation (**c**).

The read quality was high and the assembled genome had a size of 4,264,659 bp by 693× sequencing depth that was constructed by de novo assembly using the SPAdes method. The information from genome construction showed that the genome contained 56 contigs, with an average contig length of 257,831 bp, and the GC content was 46%. The genome presented a completeness of 99.57%. No prophages or foreign DNA fragments were detected, indicating that the genome assembly was free from contamination. The genome sequence of *Bacillus paralicheniformis* NNS4-3 was deposited in the National Center of Biotechnology Information (NCBI) database under the accession number JBDLPF010000000. The protein-coding sequences (CDSs) were annotated by Rapid Prokaryotic Genome Annotation (Prokka) version 1.14.6 including biosynthetic gene clusters (BGCs) of secondary metabolites, which were annotated by Antibiotics and Secondary Metabolite Analysis Shell (antiSMASH) version 7.0. The prediction of antimicrobial-resistant genes localized in the genome was annotated by the Comprehensive Antibiotic Resistance Database (CARD). The annotation systems were visualized by Proksee. The analyzed genome was presented with annotations by a circular map with a number of different functions of genes that were classified and expressed by various colors (Figure 7a). The 4288 genes were annotated by Prokka and there were 4195 CDSs, 12 rRNA, 1 tmRNA, and 80 tRNA in the genome. The BGCs of secondary metabolites as antimicrobial compounds were counted by antiSMASH, which provided 13 BGCs with different similarities based on orthologs in the databases. The Rapid Annotations using Subsystems Technology (RAST) method was used to predict the cellular machinery of genome information. The genes were categorized into subsystem and non-subsystem classifications. Of the total 4526 coding sequences (CDSs), 28% were categorized into subsystems and 72% into non-subsystems. The encoding proteins annotated by subsystem functionalization were 1246 genes by which 1185 genes and 61 genes were encoded to non-hypothetical and hypothetical proteins, respectively. The subsystem category distribution found 1795 features. In contrast, 3280 genes annotated by familial genes evidenced in the database were non-subsystem. There were 1662 genes encoding non-hypothetical proteins and 1618 genes encoding hypothetical proteins (Figure 7b). The taxonomy of NNS4-3 was predicted by a genome-based sequence and analyzed by the service of the Type (Strain) Genome Server (TYGS). NNS4-3 had the closest relation to *Bacillus paralicheniformis* KJ-16. The pairwise comparison calculated by the Genome BLAST Distance Phylogeny (GBDP) method against the genomes in the TYGS database showed a similarity score of 89.4% (95% CI, 86.1–92.0%), 73.8% (95% CI, 70.8–76.7%), and 89.5% (95% CI, 86.7–91.8%) as *d*_0_, *d*_4_, and *d*_6_, respectively (Figure 7c). One primary safety evaluation for using NNS4-3 in future healthcare products is antibiotic susceptibility, in compliance with FDA regulations. We investigated antimicrobial resistance genes (ARGs) using the prediction of the resistance gene identifier (RGI) in the CARD database (Table S1) and confirmed antibiotic susceptibility with the disk diffusion method (Figure S1). Our key findings include the presence of the *fos*Bx1 gene, which suggests resistance to fosfomycin with moderate identity (55.56%) and coverage (111.59%) [12]. Four glycopeptide resistance genes (two of the *van*T gene in the *van*G cluster, the *van*Y gene in the *van*G cluster, and the *van*W gene in the *van*I cluster) exhibited low identity (32.26–39.39%) and moderate–high coverage (54.78–97.85%) but vancomycin susceptibility was high, indicating these genes might have low-level expression or alternative functions [13]. The *Bc*III gene, a class-A beta-lactamase, showed moderate identity (64.40%) and high coverage (97.15%), leading to ceftriaxone resistance while being inhibited by cefoxitin [14]. The *qac*J gene, conferring resistance to disinfectants via an efflux pump, had low identity (42.00%) and high coverage (98.13%). Additionally, genes *bcr*A, *bcr*B, and *bcr*C, related to bacitracin resistance, exhibited high identity (98.69–99.52%) and coverage (100.00–107.84%), consistent with the production of a bacitracin-like peptide [15,16,17]. Antibiotic susceptibility testing revealed the highest susceptibility to erythromycin, with high potency inhibition by ciprofloxacin, piperacillin-tazobactam, and imipenem. Cefoxitin, gentamicin, doxycycline, and vancomycin showed moderate to high susceptibility, while the isolate tolerated ceftriaxone at normal doses (Figure S1). These results suggest that NNS4-3 is susceptible to commonly used antibiotics in both community and hospital settings.

### 2.11. Comparison of Biosynthetic Gene Clusters of NNS4-3

**Figure 8:**
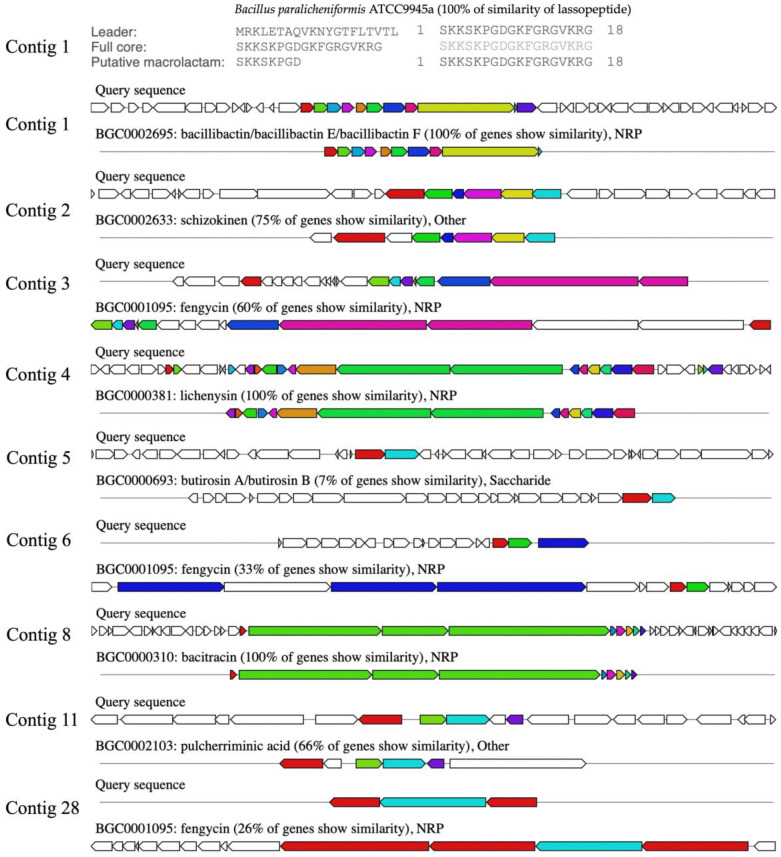
The secondary metabolites produced by BGCs that were predicted by the similar orthologs in the MIBiG database. The similarity predictions of *Bacillus paralicheniformis* NNS4-3 BGCs are presented by the arrangement of colored genes, indicating the order and function of genes in the cluster that were responsible for secondary metabolite production. The color code indicates the functional type of the predicted domain following the AntiSMASH cluster visualization.

The BGCs were predicted by genome information analysis using the antiSMASH platform. The sub-platform of antiSMASH, Minimum Information about a Biosynthetic Gene cluster (MIBiG), predicted 13 gene clusters, which were annotated and reported by similar orthologs. The biosynthetic gene cluster comparison was determined. It was found that the analyzed genome contained three clusters of ribosomally synthesized and post-translationally modified peptides (RiPPs), six clusters of non-ribosomal peptides (NRPs), one cluster of polyketides (PKs), and three clusters of others. Ten predicted BGCs of the NNS4-3 genome were matched with the known gene clusters in the database. Each gene showed different similarities. The highest similarity score indicated 100% similarity to BGCs of lasso peptide, bacillibactin, lichenysin, and bacitracin. The moderate similarity ranged from 33 to 75%, showing the BGCs of shizokinen, pulcherriminic acid, and fengycin. The lowest similarity annotated as others cluster was 7% to BGCs of butirosin (Figure 8). Predicting the BGCs using similar orthologs provided insights into exploring the genome and determining the gene products of various secondary metabolites.

**Figure 9:**
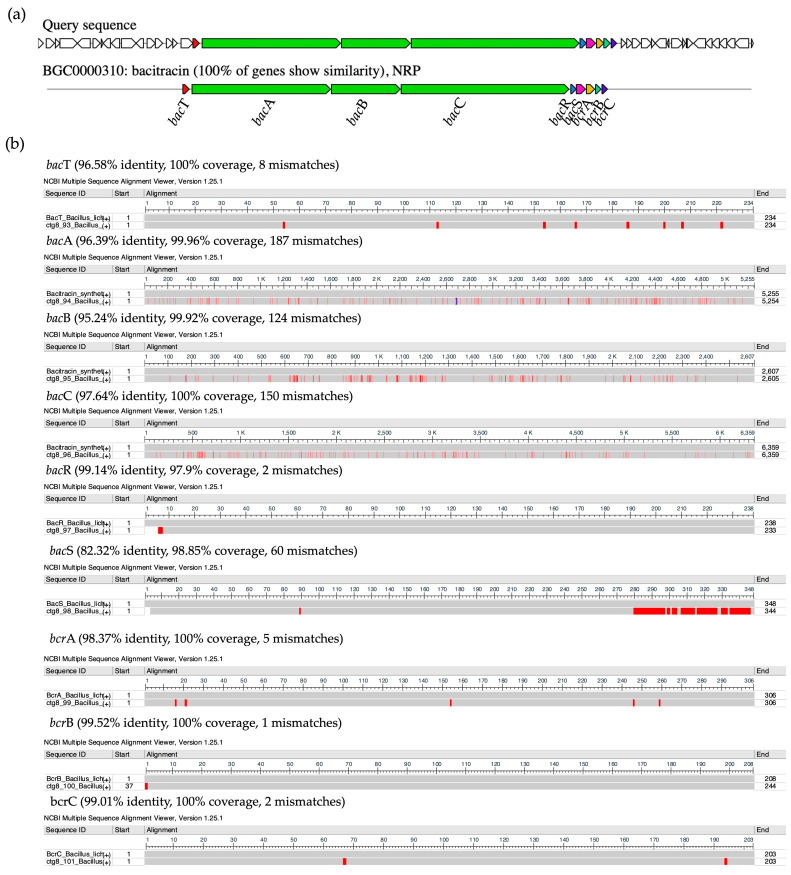
The ortholog comparison of BGCs between *Bacillus paralicheniformis* NNS4-3 and the reference *Bacillus licheniformis* ATCC 10716. The prediction showed that the coding gene of NNS4-3 displayed 100% similarity to gene orthologs that were a cluster of bacitracin-like biosynthetic genes against the reference cluster. The function of predicted domain was represented by the color code in the AntiSMASH cluster visualization system (**a**). The encoded proteins from each gene that were responsible for AMP synthesis by modular enzymes (**b**). The differences in amino acid residues of each module enzyme (below) were compared to the reference (above), which is indicated by the red color along the amino acid sequences. The amino acid sequence similarity of each coding protein is presented with identity (%), coverage (%), and number of mismatches.

The purified AMP of NNS4-3, which was determined the amino acid sequence using mass spectrometry (Lys-Leu-Leu-Lys-Asp-Leu-Phe-His-Asp-Asn), showed a sequence relevant to the amino acid sequence of bacitracin (Leu-Cys-Leu-Glu-Ile-Lys-Orn-Ile-Phe-His-Asp-Asn). AntiSMASH results showed that the gene cluster ortholog was 100% similar to the bacitracin BGC (BGC0000310) in the MIBiG database (Figure 9a). This reference gene cluster belonged to *Bacillus licheniformis* ATCC 10716 (NCBI GenBank: AF007865.2). The encoded amino acid sequences of genes in the predicted bacitracin-like BGC were compared to the bacitracin BGC in the database by pairwise alignment. Differences in encoded amino acids between each gene within the BGC were found. Bacitracin is an AMP constructed by non-ribosomal peptide synthesis via orchestrated enzymes. There were *bac*T, *bac*A, *bac*B, *bac*C, *bac*R, *bac*S, *bcr*A, *bcr*B, and *bcr*C coding sequences responsible for bacitracin synthesis that matched with 82.32–99.52% identity and 97.9–100% coverage of the query amino acid sequence of the NNS4-3 genome (Figure 9b). Interestingly, the core biosynthetic genes, including *bac*A, *bac*B, and *bac*C, which encoded the principal enzymes for bacitracin production, showed amino acid sequence identities with the NNS4-3 coding region of 96.39%, 95.24%, and 96.64%, respectively. Both AMPs shared a conserved amino acid sequence of Ile-Lys-Orn-Ile-Phe-His-Asp-Asn. This finding supported the relevance of the amino acid sequence of NNS4-3 AMP to the amino acid sequence of bacitracin. However, the limitation of mass spectrometry is its inability to distinguish between isoleucine (Ile) and leucine (Leu), which have the same molecular weight.

## 3. Discussion

Antimicrobial resistance is a global issue that healthcare providers, governments, and private sectors are spotlighting. MRSA poses an infection risk that the WHO is concerned about and created an action plan to solve [18]. In all ages of patients, especially the elderly and children, who are vulnerable to infection, this requires intensive treatment to cure [19,20]. Mangrove sediments provide various nutrients and abundant organic matter, creating a favorable environment for microbial growth despite the high-salt and low-oxygen conditions. There are natural resources for organism diversity, including microbes, such as actinobacteria, proteobacteria, firmicutes, planctomycetes, bacteroidetes, and gamma-proteobacteria [21]. Mangrove areas in Thailand are commonly found in the southern part of the country. The report showed that microbial isolation provided 519 natural products. Of these, 210 compounds were from bacteria (mostly from streptomyces) and 309 compounds were from fungi (mostly from *Aspergillus* and *Penicillium*) [22]. Another report showed that firmicutes have the potential for bioactive discoveries.

Firmicutes microbes, particularly *Bacillus* species, have been found to produce potent antimicrobial compounds as secondary metabolites with broad-spectrum activity against harmful bacteria, including MRSA [23]. A total of 1286 *Bacillus* strains exhibiting antimicrobial activity have been reported, such as *Bacillus subtilis* (n = 348), *Bacillus amyloliquefaciens* (n = 214), *Bacillus licheniformis* (n = 114), *Bacillus circulans* (n = 89), *Bacillus thuringiensis* (n = 73), *Bacillus pumilus* (n = 61), *Bacillus velezensis* (n = 60), *Bacillus megaterium* (n = 17), *Bacillus mojavensis* (n = 17), and unidentified bacillus species (n = 293) [24]. The reports on antimicrobial production by *Bacillus paralicheniformis* are relatively low in number. The phylogenetic relation of NNS4-3 showed the closest relation to *Bacillus paralicheniformis* KJ-16 based on genome comparison. *Bacillus paralicheniformis* has been formerly identified as *Bacillus licheniformis*. Molecular genetics has confirmed the difference between both species since 2015. The difference between the two species relies on distinct operons that contribute to the BGC of secondary metabolites. A previous study has described the colony morphology of *Bacillus paralicheniformis* MDJK30 cultured in a casein medium. The colonies were beige in color with a dry-textured surface and irregularly shaped borders. It was a Gram-positive rod capable of forming endospores [25]. SEM imaging of *Bacillus paralicheniformis* RP01 cultured in Luria Bertani medium displayed rod-shaped cells with endospore formation and the vegetative cells had a diameter of 2–3 μm [26]. The colony and cell morphology of *Bacillus paralicheniformis* NNS4-3 were similar to those of the aforementioned strains. Nonetheless, *Bacillus paralicheniformis* NNS4-3 exhibited a distinct characteristic of brick-red pigment production in its colonies, which could suggest a different phenotypic expression.

*Bacillus paralicheniformis* NNS4-3 presented BGCs responsible for the production of antimicrobial compounds, such as lasso peptide, bacillibactin, lichenysin, bacitracin, and fengycin, which was consistent with *Bacillus paralicheniformis* strains MDJK30 and ATCC9945a [25,27]. *Bacillus paralicheniformis* has the capability to produce fengycin, paralichenicidin, and bacitracin, whereas *Bacillus licheniformis* is unable to produce these compounds [28]. *Bacillus* species have different operons with the homology identity of biosynthesis enzymes of fengycin ranging from 62% to 74%. This supports the evidence of divergence from the same ancestor. The bacillibactin BGC was predicted to have 100% similarity ortholog. Bacillibactin is synthesized by the *dhb* gene cluster. The detected operon, *dhb*ACEBF, was involved in the 2,3-dihydroxybenzoate (dhb) biosynthesis pathway with a multi-enzyme complex that has been reported in *Bacillus paralicheniformis* strain BP9 [29]. Lichenysin has been reported in a previous study. Genome sequence analysis of several strains of *Bacillus licheniformis* revealed the presence of *lic*A, *lic*B, *lic*C, and *lic*TE, which were responsible for lichenysin biosynthesis. The differences in the DNA sequence of these genes contributed to the difference in the carbon chain length of lichenysin [30]. Fengycin was a non-ribosomal synthesized peptide that exhibited antifungal activity. The cluster contributing to the fengycin synthesis was composed of *fen*A-E genes, which have been found in the genomes of *Bacillus paralicheniformis*, *Bacillus amyloliquefaciens*, *Bacillus subtilis* and *Bacillus velezensis* [31]. The BGC of bacitracin found in the NNS4-3 genome has also been identified in *Bacillus paralicheniformis*, *Bacillus sonorensis*, *Bacillus thuringiensis*, and *Bacillus cereus*. This BGC contained *bac*A, *bac*B, *bac*C (bacitracin synthetase), *bac*T (thioesterase), *bac*R and *bac*S (two-component regulatory system), and *bac*E (ABC transport gene as *bcr*A, *bcr*B, and *bcr*C). The bacitracin synthetase in *Bacillus paralicheniformis*, *Bacillus sonorensis*, *Bacillus thuringiensis*, and *Bacillus cereus* showed high similarity to genes for subpeptin synthesis from *Bacillus subtilis*, suggesting that horizontal gene transfer (HGT) of these genes might have occurred among these species.

The amino acid sequences of bacitracin synthetase across different strains exhibit sequence divergence but they share conserved substrate domains, indicating functional conservation of these enzymes [25,32]. The self-resistance mechanism to bacitracin has been reported. The *bac*R and *bac*S genes regulate the bacitracin resistance along with the ABC transporter genes (*bcr*A, *bcr*B, and *bcr*C), which protect it against bacitracin damage during its production [33]. The other BGCs, such as butirosin, schizokinen, and pulcherriminic acid, show low to moderate similarity in their orthologs (7–75% similarity). Further investigation and verification of these BGCs and their functions are needed. The bacitracin-like AMP purified from NNS4-3 revealed a structure related to the familial structure of AMPs derived from *Bacillus* species. Bacitracin, lichenin, subpeptin, and subpeptin-like AMP have been reported to have similar amino acid sequences in the part of -Ile-Phe-His-Asp-Asn [32,34,35]. NNS4-3 AMP also contained the same conserved amino acid sequence as the aforementioned AMPs. However, mass spectrometry could not discriminate between the isoleucine and leucine amino acids. Therefore, the investigated AMP from NNS4-3 exhibited a 50% similarity to bacitracin. The BGCs for paralichenicidin and amyloliquecidin have been reported to be present in some strains of *Bacillus paralicheniformis*. However, these BGCs were not found in the genome of *Bacillus paralicheniformis* NNS4-3. This indicated that the BGCs of secondary metabolites were diverse among subspecies [25].

The further utilization of bacterial isolate as a component of the products in the healthcare industry requires an antibiotic susceptibility profile for safety data requirements, as regulated by the FDA. *Bacillus paralicheniformis* NNS4-3 still exhibits high susceptibility to many antibiotics. In contrast, 24 strains of *Bacillus paralicheniformis* and 74 strains of *Bacillus licheniformis* have been reported to resist erythromycin and clindamycin due to the presence of the *erm*D gene and *spe*G gene [36]. Conversely, these two genes were not found in the in silico prediction of the NNS4-3 genome and susceptibility tests have confirmed its susceptibility to erythromycin. Regarding bacitracin resistance, almost all *Bacillus paralicheniformis* strains possess these genes for self-defense during the production of bacitracin as a secondary metabolite for competitive situations in natural environments [33]. Several antibiotic-resistance genes were found in the genome of NNS4-3, including genes encoding resistance to penicillin, aminoglycosides, tetracycline, and vancomycin. However, susceptibility testing verified that NNS4-3 was susceptible to those aforementioned antibiotics. The results were supported by previous studies. Nine strains of *Bacillus paralicheniformis* also exhibited a susceptible profile against the mentioned antibiotics [37,38]. The antibiotic susceptibility profile of NNS4-3 showed similarities with three strains of *Bacillus velezensis*, which were susceptible to ciprofloxacin but resistant to cephalosporins (cefoxitin, cefotaxime, and ceftriaxone) [39]. Nevertheless, NNS4-3 demonstrated resistance to ceftriaxone while it was susceptible to other cephalosporins. The antibiotic susceptibility of NNS4-3 was within acceptable limits for conventional treatment, indicating that this strain showed a low risk for pathogenicity. Previous studies have reported different MICs for bacitracin from *Bacillus paralicheniformis*. In the early stationary phase of the growth curve (48–72 h of incubation), many *Bacillus* species produce the maximum potency of the antimicrobial peptide [23]. The early stationary phase of NNS4-3 growth showed the maximum antimicrobial activity at 48 h. The purification procedures together with the bioassay guide provided the achievement of AMP purification. The antimicrobial peptide-producing *Bacillus* genus was supported by much of the reported literature [24].

The MIC value of the purified AMP obtained from *Penicillium sclerotiorum* was 250 μg/mL for the methicillin-susceptible *S. aureus* (MSSA) strain SA1911 and 1911B and it was 125 μg/mL for the MRSA strain ATCC 35139 [40]. In another study, the MIC value of bacitracin was 64 μg/mL in the MSSA strain RN4420 and 32 μg/mL in the MRSA strain MW2 [41]. The literature search could support the observed difference in MIC between MSSA and MRSA strains. In our study, the MIC values of NNS4-3 AMP were different against MSSA and MRSA with lower MICs (1 µg/mL–16 µg/mL) than those reported in previous works. Interestingly, MRSA strains were more susceptible to the NNS4-3 AMP than the MSSA strain.

The sensitivity of bacitracin derived from *Bacillus paralicheniformis* UBBLi30 was studied against several conditions for 1 h. The stability result has been reported, showing that bacitracin was stable at up to 100 °C and across a wide range of pHs (1–11) with 100% remaining activity. The activity was lost when the pH reached 14. Autoclaving for 15 min diminished the activity of the AMP to 80%. In addition, the AMP could resist digestion by proteolytic enzymes, such as proteinase K, trypsin, and pepsin [42]. The NNS4-3 AMP showed similar stability against temperature treatment, proteolytic enzyme degradation, surfactant treatment, and pH variation to the reported bacitracin for 1 h of treatment. Moreover, our study extended the treatment duration to 6 and 12 h, which is longer compared to the previously reported study. The NNS4-3 AMP showed thermal resistance up to 80 °C and maintained robust activity across a wide range of physiological pHs for 12 h. Notably, temperatures above 80 °C for more than 6 h destroyed the peptide activity in a time- and temperature-dependent manner. Autoclaving reduced the activity of the NNS4-3 AMP by half within 15 min and all activity was diminished after 30 min. These thermal instability phenomena could be attributed to changes in the secondary structure of the AMP from alpha-helix and beta-sheet to a random-coiled structure due to denaturation. This structural change reduced the ability of AMP to destabilize bacterial membranes, resulting in a loss of antimicrobial activity [43]. The NNS4-3 AMP exhibited tolerance to proteinase K, trypsin, and alpha-chymotrypsin. The AMP was composed of one phenylalanine residue as an aromatic amino acid and two lysine residues, which served as specific cleavage sites for proteinase K and alpha-chymotrypsin targeting the aromatic amino acid and trypsin targeting lysine, respectively [44]. However, the activity of the AMP remained above 90% due to the short length of the peptide and its enantiomeric structure containing D-amino acids, which could not be cleaved by the aforementioned enzymes [43]. Nonetheless, the mass spectrometry technique employed in our study was unable to determine the stereochemistry of the amino acid residues. The activity of NNS4-3 AMP could be reduced by SDS, whereas Triton X-100 did not affect the antibacterial activity of the AMP. Surfactants could form micelle structures above a critical micelle concentration and subsequently interact with lipid bilayers, such as bacterial cell membranes. The reduced activity of the NNS4-3 AMP in the presence of SDS could be proposed to occur due to the binding of the positively charged NNS4-3 AMP to the negatively charged micelles formed by SDS, which mimicked the interaction with intact bacterial cell membranes. This interaction was formed by electrostatic forces. Subsequently, hydrophobic interactions played a key role in facilitating the interaction of the hydrophobic part of the AMP with the bacterial cell membranes or SDS micelles. It could be proposed that the ability of the AMP to destabilize bacterial cell membranes caused cell disruption. In the SDS environment, the AMP could bind to SDS micelles instead of bacterial cell membranes; this reduced the availability of AMP molecules to interact with bacterial cells. Consequently, a reduction in AMP activity was observed in the presence of SDS. In contrast, the non-ionic micelles of Triton X-100 forming uncharged micelle surfaces had a lower impact on AMP binding. Therefore, AMP molecules would be able to fully interact with bacterial cells in the presence of Triton X-100 [45].

The mechanisms by which AMPs inhibit bacterial growth can be categorized into two types. The first is the receptor-mediated mechanism. Well-studied AMPs, like nisin and polymyxin, exert their activity by binding to lipid II and lipopolysaccharide on the bacterial cell wall, respectively [46]. Likewise, bacitracin disrupts bacterial cell wall synthesis by binding to lipid-P-P during the dephosphorylation of C55-isoprenyl pyrophosphate [47]. The second mechanism is non-receptor-mediated, which is more commonly reported for AMPs. The physical interaction plays an important role in the binding of cationic AMPs to the negatively charged surface of bacterial cell membranes due to the presence of phospholipids, teichoic acids, and lipopolysaccharides. The surfaces of Gram-positive bacteria, such as *S. aureus* and MRSA, contain teichoic acids, resulting in negatively charged cell membranes. This leads to an initial electrostatic attraction between the cationic AMPs and the bacterial cell membranes [48]. After the electrostatic interaction, hydrophobic interactions between AMPs and bacterial cell membranes occur. This leads to the destabilization of the bacterial cell membrane and subsequent membrane disruption. This is the mechanism by which AMPs exert their antimicrobial activity [11]. The well-defined models of molecular action for AMPs via the non-receptor-mediated mechanism are the barrel-stave, toroidal, and detergent-like carpet models, which have been described for alamethicin, lacticin Q, and aurein, respectively [49]. SEM micrographs revealed that the NNS4-3 AMP contributed to pore formation, cell membrane destabilization, and membrane breakdown, which were similar to the SEM images of vancomycin, which acts as a membrane-disrupting compound. These actions could be attributed to the 10-amino-acid length of the NNS4-3 AMP, which was composed of lysine, aspartic acid, and leucine residues in its structure. This resulted in cationic, anionic, and hydrophobic moieties, respectively. Additionally, the secondary structure of NNS4-3 AMP was confirmed by CD spectra. The beta-sheet conformation with an antiparallel direction plays an important role in pore formation on the bacterial membrane. A previous study reported the beta-sheet conformation with an antiparallel direction of synthetic AMPs facilitated penetration and pore formation on the cell membranes of both Gram-positive and Gram-negative bacteria by physical interaction [50]. Furthermore, the converted structure conformation from a random coil conformation to an alpha-helix conformation of the NNS4-3 AMP when interacting with SDS micelles supported its action on bacterial cell membranes [51]. Another crucial aspect determining an antibiotic’s effectiveness is its bactericidal rate, which refers to the rate at which it eliminates targeted bacteria. A faster bactericidal action typically leads to a quicker reduction in the bacterial load within the patient. This study suggested that at 4× MIC levels, the NNS4-3 AMP completely eradicated the *S. aureus* TISTR 517 within 16 h, while its bactericidal activity against the MRSA strain 2468 was observed within 8 h.

Killing kinetics revealed that the NNS4-3 AMP exhibited a mode of action with time- and concentration-dependent killing characteristics against the MSSA and MRSA strains. Most reported AMPs have been defined to exhibit modes of action that are dependent on both concentration and time. For alpha-helical cationic antimicrobial peptides, such as nisin and pleurocidin, their activity exhibits concentration dependence whereby a critical concentration is required to disrupt the cell membrane and cell wall of the treated bacteria via the previously described mechanisms. In addition to the concentration-dependent effect, AMP also exhibited a time-dependent killing effect, which involved slow pore formation on the bacterial cell membrane or the gradual accumulation of AMP to disrupt intracellular targets leading to cell death [52,53,54].

The suggestion for further study is to determine the mechanisms of action at the molecular level of the NNS4-3 AMP. Verification of the predicted genes is encouraged to confirm the production process of the AMPs that were annotated in the genome, as they might contain low to moderate similarity to known orthologs. This potentially leads to the discovery of new gene products. *Bacillus paralicheniformis* NNS4-3 could be a promising candidate as a source of AMPs against MRSA strains. Further evaluation of the AMP toxicity and safety is also required for clinical use. The insights obtained from its genomic information could support new approaches in synthetic biology to combat antimicrobial resistance.

## 4. Materials and Methods

### 4.1. Sample Collection and Bacterial Isolation

Mangrove sediment was collected from the Pak Banang District, Nakhon Si Thammarat, Thailand. The sediment samples were collected at a depth of 10–15 cm from the surface. The samples were stored in clean polyethylene bags and packed into an ice box. In total, 10 g of sediment was transferred into a sterile flask and aseptically diluted with 90 mL of sterile 0.85% NaCl solution (RCI Labscan Ltd., Bangkok, Thailand). The sediment suspension was agitated in a shaking incubator at 150 rpm for 30 min before being incubated at 60 °C for 30 min to select spores during the bacterial dormant stage. The samples were diluted 10-fold serially up to 10^−6^. Each dilution (100 µL) was spread on MH agar, ZM agar, and 1.5% NaCl-supplemented SC agar (Titan Biotech Ltd., Rajasthan, India). The seeded plates were incubated at 30 °C for 7 days for colony growth [55].

### 4.2. Screening of Antibacterial Activity by the Soft Agar Overlay Method against the MRSA Strain

A single colony of the isolate was placed on MH agar and incubated at 30 °C for 3 days. The MRSA strain 2468 was used as a test strain. It was subcultured on MH agar and incubated at 37 °C for 18 h. The suspension of MRSA was prepared by dispersing a single colony in a 0.85% sterile NaCl solution and adjusting the turbidity to be equivalent to 0.1 optical density (OD) at 625 nm (Genesys 20, Thermo Scientific, Waltham, MA, USA). Additionally, 1 mL of MRSA suspension (1 × 10^8^ CFU/mL) was homogeneously dispersed in 9 mL of molten 0.7% agar containing MH medium before overlaying on the bacteria-seeded plates. The soft agar overlaid plates were incubated at 37 °C for 24 h. The antimicrobial compound-producing isolates were observed by the appearance of an inhibition zone [56].

### 4.3. Verification of Antibacterial Activity by the Agar Well Diffusion Method

The active isolates from the agar overlay assay were inoculated in broth. The types of broth media were the same as those used during the initial isolation. The liquid cultures were incubated at 30 °C with shaking at 150 rpm for 18 h. The optical density of the starter culture was adjusted to a turbidity of 0.1 OD at 625 nm with 0.85% sterile NaCl solution before transferring 1 mL of the culture into 49 mL of fresh broth. The inoculum was then incubated at 30 °C and 150 rpm for 3 days. The cell-free supernatant (CFS) was collected by centrifugation at 10,000× *g* at 4 °C for 15 min and filtered using a 0.2 µm sterile cellulose acetate syringe filter (Sigma-Aldrich, Warren, MI, USA). The antibacterial activity of the isolates was studied using *S. aureus* TISTR 517, obtained from the Thailand Institute of Scientific and Technological Research (TISTR), Thailand. The three strains of MRSA (strains 142, 1096, and 2468) were kindly provided by the medical technology laboratory of the School of Allied Health Sciences, Walailak University, Thailand. The pathogenic bacteria were incubated at 37 °C for 18 h on MH agar. The microbial indicators were prepared to a turbidity equivalent to 0.1 OD at 625 nm before being spread on MH agar. The CFS of 100 µL was aseptically transferred to 9 mm diameter wells, followed by an incubation period at 37 °C for 18 h. Cefoxitin (30 µg) and vancomycin (30 µg) (Sigma-Aldrich Co., St. Louis, MO, USA) were employed as positive controls. The experiment was conducted in triplicate and the mean ± SD of the diameters of the inhibition zones was measured [57].

### 4.4. Studies on the Production Kinetics of Antimicrobial Compounds

The preculture of the most active isolate (NNS4-3) was prepared with an equivalent OD of 0.1 at 625 nm and then inoculated at a concentration of 2% in 50 mL of ZM broth. The sample was incubated at 30 °C with shaking at 150 rpm for 7 days. The OD at 625 nm of the inoculum was measured and the CFS was collected at 0, 4, 8, 12, 16, 20, and 24 h on the first day of incubation and then once a day until the seventh day of incubation. The bacterial growth was observed by measuring OD at 625 nm and the collected CFS at all time points was evaluated for antibacterial activity using the agar well diffusion assay against *S. aureus* TISTR 517 and three strains of MRSA. The experiment was performed in triplicate. The statistical analysis was used to compare the antibacterial activity of the CFS at each incubation time, determined by the Student’s *t*-test at a *p*-value < 0.05. The kinetics of the antimicrobial compounds’ production curve with mean ± SD were presented [58].

### 4.5. Purification of Anti-MRSA Components

One-day-old cultures of NNS4-3 colonies were suspended in 0.85% sterile NaCl solution. The bacterial suspension was prepared to have a turbidity equivalent to 0.1 OD at 625 nm. The OD-adjusted bacterial suspension was used to prepare a 2% inoculum in 200 mL of ZM broth in a 1 L sterile Erlenmeyer flask. The 1 L total culture was incubated at 30 °C and 150 rpm for 48 h. The CFS was collected as described in the previous experiment. Ammonium sulfate at 25%, 50%, and 75% saturation levels were prepared by the stepwise addition of ammonium sulfate to the collected CFS. The precipitates from each saturation were collected by centrifugation at 18,000× *g* at 4 °C for 15 min. The collected precipitates were dissolved in 0.1% TFA in water before being desalted using a dialysis bag with a 3.5 kDa molecular weight cut-off membrane (SnakeSkin membrane, Pierce, Rockford, IL, USA). The dialysis was performed in 0.1% TFA in water at 4 °C for 16 h. Each dialyzed fraction was tested for antibacterial activity using an agar well diffusion assay against the MRSA strain 2468. Subsequently, the fraction exhibiting antibacterial activity was subjected to RPC using an Inertsil ODS-3 C18 column (4.6 × 250 mm; GL Sciences, Tokyo, Japan) as a stationary phase. A gradient elution of mobile phase A (0.1% TFA in water) and mobile phase B (0.1% TFA in 70% acetonitrile; ACN) at a flow rate of 1 mL/min was used for the separation of the antibacterial components. The gradient elution was performed as follows: 0.0% mobile phase B for 25 min, 0.0% to 60% mobile phase B for 120 min, and 100% mobile phase B for 40 min. The peak was detected at 214 nm. Each fraction of 1 mL was collected from the chromatography and subsequently evaporated in a speed-drying vacuum concentrator (RVC 2-25 CDplus, Martin Christ, Osterode am Harz, Germany). The dried components were dissolved in purified water to a volume of the same collected fraction and then tested for antibacterial activity using agar well diffusion assay against the MRSA strain 2468. The total volume of active fractions from each purification step was measured. The collected active fractions were dried by lyophilization (Gamma 2-16 LCSplus, Martin Christ, Osterode am Harz, Germany) before their weights were determined. The dried components were reconstituted with sterile purified water to the same volume as the volume before drying. The reconstituted fractions were prepared at varying concentrations through 2-fold dilution and assessed for antibacterial activity using agar well diffusion assay against the MRSA strain 2468. The arbitrary activity of each active fraction was calculated by taking the final dilution to the power of 2 that showed an inhibition zone and multiplying it by 10.

### 4.6. Sodium Dodecyl Sulfate-Polyacrylamide Gel Electrophoresis (SDS-PAGE) and Agar Overlay Assay

SDS-PAGE was used to assess the purification efficacy and determine the active protein band with an estimated molecular weight [59]. The purified fractions obtained from each purification step were subjected to a 15% polyacrylamide gel. A sample weight of 5 µg was loaded in each well. The electrophoresis was performed at 120 V. The electrophoresed gel was cut into two parts. One part of the gel was stained with coomassie brilliant blue G-250 to visualize the protein bands. The other part was used to determine the location of the protein band exhibiting antibacterial activity. The gel was fixed with a mixture of 25% ethanol and 5% glacial acetic acid for 1 h and then washed with purified water for 3 h. The soft agar overlay assay using the MRSA strain 2468 as the test strain was conducted following the previous method. After incubating at 37 °C for 18 h, the overlaid gel was monitored for the position of the inhibition zone.

### 4.7. Peptide Sequencing by Mass Spectrometry and De Novo Peptide Sequencing

Peptide sequencing was conducted following the previously reported methods [60]. The purified sample was analyzed by utilizing an UltiMate 3000 liquid chromatography (LC) system combined with high-resolution mass spectrometry (MS) (Thermo Fisher Scientific Inc., Waltham, MA, USA). The peptide was dissolved in 0.1% formic acid and 1% ACN before being injected into a reversed-phase UHPLC column (4.6 × 30 mm; C18 column Hypersil Gold, Thermo Fisher Scientific Inc., Waltham, MA, USA). The injected sample was separated by the gradient elution from buffer A (0.1% formic acid) to buffer B (0.1% formic acid in ACN) in 40 min at a flow rate of 300 µL/min. The separated peptides were ionized using an electrospray ionization source with a capillary voltage of 3.2 kV at 300 °C. The de novo sequencing was performed to determine the peptide sequences along with LC-MS data in the mode of full mass scanning. The MS parameters for detecting the peptide fragmentation mass were as follows: the resolution was 120,000, the automatic gain control (AGC) target was 1 × 10^6^, the maximum injection time was 100 ms, and the scanning range was m/z 400–2200. The result of the full MS scan was processed using Freestyle software (version 1.4) (Thermo Fisher Scientific Inc., Waltham, MA, USA) for peak identification. The identified peaks from the parallel reaction monitoring system were analyzed for fragmentation by the second mass spectrometry (MS2). The MS2 parameters were as follows: the resolution was 30,000, the AGC target was 1 × 10^6^, the maximum injection time was 100 ms, and the isolation window was *m*/*z* 1.4. The collected mass data were used to predict the amino acid sequences using Peak Studio X (Bioinformatics Solutions Inc., Waterloo, ON, Canada). The predicted peptide sequences were included in the analysis if the ALC score was above 70%. The prediction of physicochemical parameters was performed using ProtParam on the Expasy server [61].

The secondary structure of the purified AMP was determined by CD spectroscopy (JASCO Corporation, Tokyo, Japan) with the wavelength scanning from 190 to 250 nm. The purified AMP (1 mg/mL) was dissolved in purified water and 50 mM SDS [62]. The CD spectra were analyzed to determine the secondary structure and calculated the component of the secondary structure in the AMP using the BeStSel method via a web-based service [63]. The helical wheel of the purified AMP was used to determine its alpha-helical structure and amino acid arrangement, which were facilitated by HELIQUEST [64]. Molecular modeling was employed to provide a three-dimensional conformation of the AMP. The molecular model of the AMP was predicted by PEP-FOLD4 for visualizing and determining its molecular surface area [65].

### 4.8. Determination of Minimum Inhibitory Concentration (MIC) and Minimum Bactericidal Concentration (MBC) of the Antimicrobial Compound

The MIC and MBC of the purified peptide of NNS4-3 were determined against various strains of *S. aureus*. The broth microdilution method followed the guidance provided by the Clinical and Laboratory Standards Institute (CLSI) [66]. Pathogenic bacteria, such as *S. aureus* TISTR 517 and MRSA strains 142, 1096, and 2468, were used as test strains and cultured on MH agar at 37 °C for 18 h. A single colony of each tested bacterium was suspended in 0.85% NaCl until its turbidity reached an OD of 0.1 at 625 nm (1 × 10^8^ CFU/mL). The bacterial cells were diluted to 5 × 10^6^ CFU/mL using cation-adjusted Mueller Hinton broth (CAMHB). The diluted cell suspension (10 µL) was transferred to each well of a 96-well plate. With a total volume of 100 µL in each well, the purified AMP was added to each well to obtain final concentrations between 0.125 and 64 µg/mL. Standard antibiotics (vancomycin and cefoxitin) and antibiotic-free samples were used as positive and negative controls, respectively. The 96-well plate was incubated at 37 °C for 24 h. The experiment was carried out in triplicate for each strain. The MIC was defined as the lowest concentration of the components that showed no observable growth of bacteria. Subsequently, 100 µL of each dilution was spread on MH agar and then incubated at 37 °C for 24 h. The MBC was identified as the lowest concentration at which no bacterial colony growth was observed on the plate.

### 4.9. Scanning Electron Microscopy (SEM) of AMP-Treated Cells

The treatment effect of NNS4-3 AMP on tested bacterial cells was investigated by observing morphological changes under SEM. *S. aureus* TISTR 517 and MRSA strain 2468 were precultured in MH broth for 18 h before being centrifuged to harvest the cell pellet. Cell pellets of both strains were washed with 0.85% sterile NaCl solution 3 times to remove exopolysaccharide. The washed cells were then dispersed in CAMHB to adjust cell turbidity to 0.1 OD at 625 nm. The prepared cells were diluted to 5 × 10^5^ CFU/mL with CAMHB before being treated with NNS4-3 AMP. The bacterial cells were incubated with 1× MIC of NNS4-3 AMP for 18 h. The sample for SEM micrography was prepared by fixing the bacterial sample in 2.5% glutaraldehyde in 0.1 M phosphate buffer pH 7.2 for 24 h before ethanol dehydration of the sample. The complete ethanol removal from the prepared sample was conducted by a critical point drying machine (Quorum Technologies Ltd., Lewes, East Sussex, UK). The dehydrated sample was coated with gold using a sputter coater machine and the micrograph images were displayed by SEM at 20,000× magnification [60]. The morphological changes of bacterial cells treated with AMP were compared to those treated with 1× MIC of vancomycin and cefoxitin.

### 4.10. Time-Kill Kinetics of the NNS4-3 AMP

*S. aureus* TISTR 517 and MRSA strain 2468 were adjusted to a cell density of 5 × 10^5^ CFU/mL with CAMHB for the initial treatment. The AMP concentrations were then prepared by diluting them to 1×, 2×, and 4× MIC using CAMHB while peptide-free CAMHB served as the non-treatment control. The reactions were conducted at 37 °C, followed by spreading the total volume onto MH agar at specific time intervals (0–24 h). Subsequently, the spread plates were incubated at 37 °C for 24 h before counting the colonies. The bacterial reduction trends were presented with a log10 scale of viable cells (logCFU/mL) versus treatment duration. The bacterial cell reduction was determined within 24 h. Each experiment was conducted in triplicate on the 96-well plates [67]. The presence of significant differences (*p*-value < 0.05) was assessed by two-way ANOVA, followed by the post hoc Tukey’s test for conducting multiple comparisons between treated and non-treated samples at each time interval.

### 4.11. Stability Studies of the NNS4-3 AMP

The stability of the NNS4-3 AMP was investigated under exposure to various conditions, including exposure times of 1, 6, and 12 h. The AMP was dissolved in sterile purified water and adjusted to a final concentration of 10 µg/mL. The sensitivity of the AMP to temperatures of 60, 80, and 100 °C as well as autoclaving at 121 °C for 15 and 30 min was evaluated. The sensitivity of the AMP to proteolytic enzymes with different digesting characteristics was studied by incubating with a concentration of 1 mg/mL of proteinase K, trypsin, and α-chymotrypsin (Sigma-Aldrich, Warren, MI, USA). Surfactant compatibility of the AMP was examined by exposing the sample to 1% SDS and 1% Triton X-100 (AppliChem GmbH, Darmstadt, Germany). The propensity of the AMP for acid and base hydrolysis was examined by adjusting the pH of the solution to 1.2, 4.5, 6.8, and 7.4. After incubation, the pH of the AMP solution was neutralized to 7.4 before being assessed for antimicrobial activity. Following all treatments, the antibacterial assay using agar well diffusion against the MRSA strain 2468 was conducted in triplicate. The stability profiles are presented as the percentage of the activity relative to the untreated conditions (mean ± SD) and statistical significance was determined by the Student’s t-test at a *p*-value < 0.05.

### 4.12. Bacterial Morphology Characterization

NNS4-3 was cultivated on ZM agar for 1 and 3 days to observe colony morphology by obtaining vegetative cells and spores, respectively. Gram staining and malachite green spore staining were performed to determine the vegetative cell morphology and spore-forming capability under a light microscope (Carl Zeiss, Oberkochen, Germany) [68,69]. SEM micrography (Carl Zeiss, Oberkochen, Germany) was employed to examine the bacterial and spore morphology. The preparation of samples for SEM micrography followed the procedures mentioned in the previous method.

### 4.13. Whole Genome Sequencing and Bioinformatic Analysis

The chromosome of NNS4-3 was extracted before sequencing by Illumina Hiseq (PE150 mode, Illumina, San Diego, CA, USA) using the service of U2Bio Co. Ltd. (Seoul, Republic of Korea). The processes of the data cleaning of raw reads and genome assembly were performed using the Galaxy Australia platform version 24.0 [70]. The raw reads were quality-checked by FastQC version 0.12.1 before and after trimming the adapters [71]. The raw reads were trimmed by Fastp version 0.23.4 to filter out the reads with a base length below 30 bases [72]. The bacterial genome was assembled by Shovill version 1.1.0 using SPAdes version 3.14.1 as the assembler [73]. The quality and genome parameters of the assembled genome were evaluated by QUAST version 5.2.0 [74]. The genome completeness and contamination of the sequences in the genome were investigated by CheckM version 1.0.18 [75]. The known genes were annotated, and the cellular machinery was predicted by Prokka version 1.14.6 and RAST, respectively [76,77]. The prediction of BGCs for antimicrobial agents was carried out by antiSMASH version 7.0 [78]. The predicted secondary metabolites from the NNS4-3 BGCs were compared to the reported reference BGCs in the database that were connected to MIBiG version 3.1 and GenBank in the NCBI database [79,80]. The insights into the bacitracin-like BGC were investigated by comparing the differences in the amino acid sequences of the encoded proteins by the Multiple Sequence Comparison by Log-Expectation (MUSCLE) algorithm in MEGA X software version 10.1.8 [81]. The NCBI Multiple Sequence Alignment (MSA) viewer version 1.25.0 was used to determine the similarity percentage and visualize the amino acid differences in the encoded proteins compared to the reference of the bacitracin BGC. The determination of ARGs was predicted by the Resistance Gene Identifier (RGI) in the CARD database. The ARGs were presented with the highest identity and coverage to the matched sequences of organisms in the Genbank NCBI database [82]. The genomic information and insight data were visualized by the Proksee web-based service. The circular genome map, including the in-depth characterization of annotated genes and genetic information, was classified according to the different colored tracks [83]. The closest taxonomic relationship of NNS4-3 was calculated by the Genome BLAST Distance Phylogeny (GBDP) method. The similarity score of the genome-based taxonomy compared to the reference strains and the construction of the genome-based phylogenetic tree were determined by the service of the TYGS genome server [84].

### 4.14. Antibiotic Susceptibility Test of NNS4-3

The susceptibility of NNS4-3 to the standard antibiotics was studied by a disc diffusion assay [85]. The isolate was inoculated at 30 °C with shaking at 150 rpm for 24 h in ZM broth. The culture was adjusted to 0.1 OD at 625 nm before being spread onto the MH agar. The antibiotic discs (Oxoid Ltd., Hampshire, UK) of ciprofloxacin (5 µg), piperacillin (100 µg) combined with tazobactam (10 µg), imipenem (10 µg), ceftriaxone (30 µg), cefoxitin (30 µg), doxycycline (30 µg), vancomycin (30 µg), erythromycin (15 µg), and gentamicin (10 µg) were placed on the culture-seeded agar and then incubated at 30 °C for 24 h. The susceptibility tests were conducted in triplicate. The inhibition zones were presented as mean ± SD.

## 5. Conclusions

*Bacillus paralicheniformis* NNS4-3 isolated from mangrove sediment could produce the bacitracin-like AMP against MRSA. The NNS4-3 AMP exhibited potent antimicrobial activity via cell membrane disruption. The NNS4-3 AMP was stable to proteolytic enzymes and surfactants and across a wide range of physiological pH conditions. However, the stability of NNS4-3 AMP against thermal conditions above 80 °C for longer than 1 h should be considered. The genomic information suggested that the findings of several BGCs for secondary metabolites could contribute to the discovery of new antimicrobial agents. The ARGs were found in the NNS4-3 genome but the antibiotic susceptibility test confirmed that this isolate was highly susceptible to various commonly used antibiotics. However, defining the mechanisms of action at the molecular level and evaluating the toxicity and safety of the AMP would require further in vitro and in vivo studies for antimicrobial effectiveness.

## Figures and Tables

**Table 1:** The cell-free supernatant of bacterial isolates from mangrove sediment exhibited antimicrobial activity against *S. aureus* TISTR 517 and MRSA strains 142, 1096, and 2468 using the agar well diffusion method.

Isolates	Zone of Inhibition (mm ± SD; n = 3)			
	*S. aureus* TISTR 517	MRSA Strain 142	MRSA Strain 1096	MRSA Strain 2468
NNS2-1	15.41 ± 1.20	19.47 ± 1.03	19.05 ± 1.83	19.98 ± 2.07
NNS4-2	12.02 ± 0.64	12.19 ± 0.15	11.94 ± 0.53	13.12 ± 0.00
NNS4-3	14.99 ± 0.59	23.20 ± 0.67	21.25 ± 0.15	22.94 ± 0.67
NNS4-5-2	0.00 ± 0.00	20.49 ± 0.51	18.37 ± 0.96	20.07 ± 0.53
Vancomycin (30 µg)	23.37 ± 1.27	24.13 ± 1.27	24.38 ± 0.51	24.64 ± 1.02
Cefoxitin (30 µg)	35.87 ± 0.46	0.00 ± 0.00	0.00 ± 0.00	0.00 ± 0.00

**Table 2:** The purification balance sheet of antibacterial components of NNS4-3.

Purification Procedure	Volume (mL)	Total Dried Weight (mg)	Activity (AU/mL)	Total Activity (AU)	Specific Activity (AU/mg)	Purification Factor	%Yield
Crude product	984	843.30	20	19,680	23.34	1.00	100.00
Precipitation	47	132.40	80	3760	28.40	1.22	19.11
RPC	12	3.63	160	1920	528.63	22.65	9.76

**Table 3:** The MIC and MBC values of the purified AMP derived from NNS4-3. The standard antibiotics (vancomycin and cefoxitin) were used as the positive controls.

Compounds	Tested Strains	MIC (µg/mL)	MBC (µg/mL)
NNS4-3 AMP	*S. aureus* TISTR 517	16	64
	MRSA strain 142	1	4
	MRSA strain 1096	1	4
	MRSA strain 2468	1	4
Vancomycin	*S. aureus* TISTR 517	2	2
	MRSA strain 142	2	2
	MRSA strain 1096	2	2
	MRSA strain 2468	2	2
Cefoxitin	*S. aureus* TISTR 517	2	2
	MRSA strain 142	>64	ND
	MRSA strain 1096	>64	ND
	MRSA strain 2468	>64	ND

ND is not determined.

**Table 4:** The stability of the AMP against temperatures, proteolytic enzymes, surfactants, and acid-base treatments. The residual activity after treatments was reported along with the incubation times (mean ± SD).

Conditions	% Residual Activity		
	1 h	6 h	12 h
**Thermal stability**			
Non-treated sample	100.00 ± 2.41	100.00 ± 0.57	100.00 ± 0.57
Treated sample at 37 °C	103.19 ± 3.68	100.00 ± 0.00	99.61 ± 6.44
Treated sample at 60 °C	101.82 ± 2.46	95.27 ± 4.31	90.47 ± 2.98 *
Treated sample at 80 °C	97.52 ± 6.21	41.13 ± 35.62 *	0.00 ± 0.00 *
Treated sample at 100 °C	73.05 ± 1.57 *	0.00 ± 0.00 *	0.00 ± 0.00 *
Treated sample at 121 °C, 15 psi, 15 min	61.83 ± 0.74 *		
Treated sample at 121 °C, 15 psi, 30 min	0.00 ± 0.00 *		
**Enzyme stability**			
Non-treated sample	100.00 ± 1.52	100.00 ± 4.29	100.00 ± 2.62
Sample + Proteinase K (1 mg/mL)	98.66 ± 1.68	99.19 ± 1.07	99.07 ± 3.08
Sample + Trypsin (1 mg/mL)	95.57 ± 4.01	95.66 ± 2.62	93.81 ± 3.31
Sample + α-chymotrypsin (1 mg/mL)	96.65 ± 2.14	95.15 ± 6.64	97.06 ± 0.54
**Surfactant stability**			
Non-treated sample	100.00 ± 6.20	100.00 ± 0.00	100.00 ± 3.74
Sample + 1% Triton X-100	104.54 ± 4.96	102.27 + 2.93	103.40 ± 3.82
Sample + 1% SDS	87.00 ± 6.17 *	86.69 ± 5.39 *	86.25 ± 4.76 *
**Effect of pH on the stability**			
Non-treated sample	100.00 ± 6.68	100.00 ± 8.51	100.00 ± 6.89
pH 1.2	98.12 ± 2.14	95.45 ± 0.79	94.14 ± 3.12
pH 4.5	95.24 ± 3.29	99.64 ± 2.87	96.91 ± 2.99
pH 6.8	98.87 ± 2.00	95.89 ± 5.00	92.54 ± 4.14
pH 7.4	97.80 ± 1.32	94.82 ± 2.80	93.29 ± 1.89

* Significance according to Student’s *t*-test at a *p*-value < 0.05 compared to non-treated sample.

## Data Availability

Data are contained within the article.

## Supplementary Materials

The following supporting information can be downloaded at: https://www.mdpi.com/article/10.3390/antibiotics13080716/s1, Figure S1: The antibiotic susceptibility profile of NNS4-3 isolate studied by disk diffusion method. Several classes of antibiotics were used to evaluate the susceptibility by different mechanisms of bacterial growth inhibition. The results were reported by the inhibition zone; Table S1: The prediction of antimicrobial-resistant genes of genomic sequence determined by Resistance Gene Identifier (RGI) in the CARD database.
