# Supplementary material for: A Novel Bacitracin-like Peptide from Mangrove-Isolated Bacillus paralicheniformis NNS4-3 against MRSA and Its Genomic Insights
Source: Antibiotics (Basel). 2024 Jul 30;13(8):716. doi: 10.3390/antibiotics13080716 (PMC11350868; doi:10.3390/antibiotics13080716)
Supplement: Supplementary file 1 [file antibiotics-13-00716-s001.zip › antibiotics-3094103-supplementary.pdf]

A Novel Bacitracin-like Peptide from Mangrove-Isolated *Bacillus paralicheniformis* NNS4-3 against MRSA and Its Genomic Insights

Namfa Sermkaew [1,2], Apichart Atipairin [1,2], Thamonwan Wanganuttara [1,2], Sucheewin Krobthong [3], Chanat Aonbangkhen [3,4], Yodying Yingchutrakul [5], Jumpei Uchiyama [6] and Nuttapon Songnaka [1,2,*]

Table S1. The prediction of antimicrobial-resistant genes of genomic sequence determined by Resistance Gene Identifier (RGI) in the CARD database.

Antimicrobial-resistant gene	ARG family	Resistance mechanism	Identity (%)	Coverage (%)
*Fos*Bx1	fosfomycin thiol transferase	antibiotic inactivation	55.56	111.59
*qac*J	small multidrug resistance (SMR) antibiotic efflux pump	antibiotic efflux	42.00	98.13
*van*T gene in *van*G cluster	glycopeptide resistance gene cluster	antibiotic target alteration	34.50	54.78
*van*Y gene in *vanG* cluster	glycopeptide resistance gene cluster	antibiotic target alteration	36.60	97.85
*bcr*A	ATP-binding cassette (ABC) antibiotic efflux pump	antibiotic efflux	98.69	107.84
*bcr*B	ATP-binding cassette (ABC) antibiotic efflux pump	antibiotic efflux	99.52	117.31
*bcr*C	undecaprenyl pyrophosphate related proteins	antibiotic target alteration	99.01	100.00
*Bc*III	class A *Bacillus cereus* Bc beta-lactamase	antibiotic inactivation	64.40	97.15
*van*W gene in *van*I cluster	glycopeptide resistance gene cluster	antibiotic target alteration	39.39	81.77
*van*T gene in *van*G cluster	glycopeptide resistance gene cluster	antibiotic target alteration	32.26	55.20

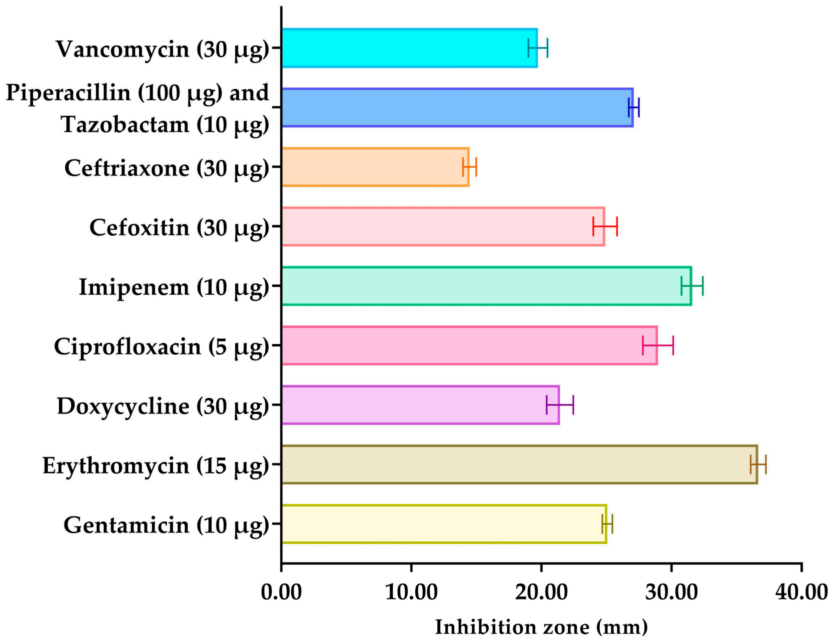

Figure S1. The antibiotic susceptibility profile of NNS4-3 isolate studied by disk diffusion method. Several classes of antibiotics were used to evaluate the susceptibility by different mechanisms of bacterial growth inhibition. The results were reported by the inhibition zone.
